# Lead poisoning after gunshot wound

**DOI:** 10.1590/S1516-31802000000300006

**Published:** 2000-05-02

**Authors:** Paulo Roberto de Madureira, Eduardo Mello De Capitani, Ronan José Vieira

**Keywords:** Lead intoxication, Gunshot wound, Joint, Sintomas de intoxicação por chumbo, Ferimentos de projétil, Articulações

## Abstract

**CONTEXT:**

Despite the absence of symptoms in the majority of patients carrying lead bullet fragments in their bodies, there needs to be an awareness of the possible signs and symptoms of lead intoxication when bullets are lodged in large joints like knees, hips and shoulders. Such patients merit closer follow-up, and even surgical procedure for removing the fragments.

**OBJECTIVE:**

To describe a patient who developed clinical lead intoxication several years after a gunshot wound.

**DESIGN:**

Case report.

**CASE REPORT:**

A single white 23-year-old male, regular job as a bricklayer, with a history of chronic alcohol abuse, showed up at the emergency department complaining of abdominal pain with colic, weakness, vomiting and diarrhea with black feces. All the symptoms had a duration of two to three weeks, and had been recurrent for the last two years, with calming during interval periods of two to three weeks. Abdominal radiograms showed a bullet lodged in the left hip, with a neat bursogram of the whole synovial capsule. A course of chelating treatment using calcium versenate (EDTACaNa_2_) intravenously was started. After the chelation therapy the patient had recurrence of his symptoms and a radical solution for the chronic mobilization of lead was considered. A hip arthroplasty procedure was performed, leading to complete substitution of the left hip.

## resumo

**CONTEXTO:**

Apesar da ausência de sintomas na maioria das pessoas que albergam projéteis ou fragmentos de projéteis de arma de fogo em alguma parte do corpo devido a acidentes passados, a ocorrência de sinais e sintomas de intoxicação por chumbo pode ser freqüente em pacientes onde o projétil está alojado em grandes articulações como joelho, quadril e ombros, merecendo maiores cuidados de seguimento clínico e procedimentos cirúrgicos de retirada.

**OBJETIVO:**

Descrever un doente que desenvolveu intoxicação por chumbo vários anos após um ferimento por arma de fogo.

**TIPO DE ESTUDO:**

Relato de caso.

**RELATO DE CASO:**

Um homem, 23 anos, branco, pedreiro, com história de uso abusivo de bebidas alcoólicas, foi atendido no prontosocorro com dor abdominal intensa em cólica, fraqueza, vômitos e diarréia com fezes escurecidas. Todos os sintomas tinham a duração de duas a três semanas, tendo sido recorrentes nos últimos dois anos, com períodos de acalmia de duas a três semanas. Radiografia de abdôme mostrou um projétil incrustrado na articulação femoral esquerda, com imagem tipo "bursograma" comprometendo toda a cápsula sinovial. Tratamento quelante usandoversenato de cálcio (EDTA CaNa_2_) endovenoso foi então iniciado. Após a terapia quelante, o paciente teve recorrência dos sintomas devido a remobilização do chumbo dos tecidos teciduais. a artroplastia da articulação foi realizada como forma de solução definitiva do problema.

## INTRODUCTION

Usually, gunshot wounds are managed conservatively because the hazard linked to surgical procedures is greater than the risk of later systemic lead intoxication.^1^ In the soft tissue and bones, the retained bullets are encased by fibrotic scar tissue with poor vascularization, avoiding lead dissolution.^2^ However, the medical literature has shown up some cases of arthropathy^3^ and systemic lead intoxication in patients whose bullets are in contact with synovial and cerebral spinal fluids.^4^ Recently, we took care of a patient who developed clinical lead intoxication several years after a gunshot wound.

## CASE REPORT

A single white 23-year-old male, regular job as a bricklayer, with a history of chronic alcohol abuse, showed up at the Emergency Department complaining of abdominal pain with colic, weakness, vomiting and diarrhea with black feces. All the symptoms had a duration of two to three weeks, and had been recurrent for the last two years, with calming during interval periods of two to three weeks. During clinical examination the patient showed facial pallor and abdominal tenderness. Laboratory blood examination showed hemoglobin = 7.4 g%; hematocrit = 23.3%; serum amylase = 123 U/L, ALT = 299 U/L; AST = 248 U/L, bilirubin = DB = 3.83; IB = 1.62; BUN = 23 mg%; serum creatinine = 0.79 mg%. An abdominal ultra-sound examination was normal, and gastric endos-copy showed erosive esophagitis.

Abdominal radiograms showed a bullet lodged in the left hip, with a neat bursogram of the whole synovial capsule (Figure). The patient gave the information that a gunshot accident had occurred seven years ago. Lead intoxication was then considered and blood samples were drawn for lead (PbB) and zinc protoporphyrin (ZPP) dosages. A urine sample was also collected for delta aminulevulinic acid (U-ALA) measurement. The following results corroborated the diagnosis of lead intoxication: PbB = 40.1 mg/dl (reference value for non-exposed population = 40 mg/dl); ZPP = 84.3 mg/dl (reference value = 75 mg/dl); and UALA = 49.2 mg/dl (reference value for non-exposed population = 6 mg/dl).

**Figure:**
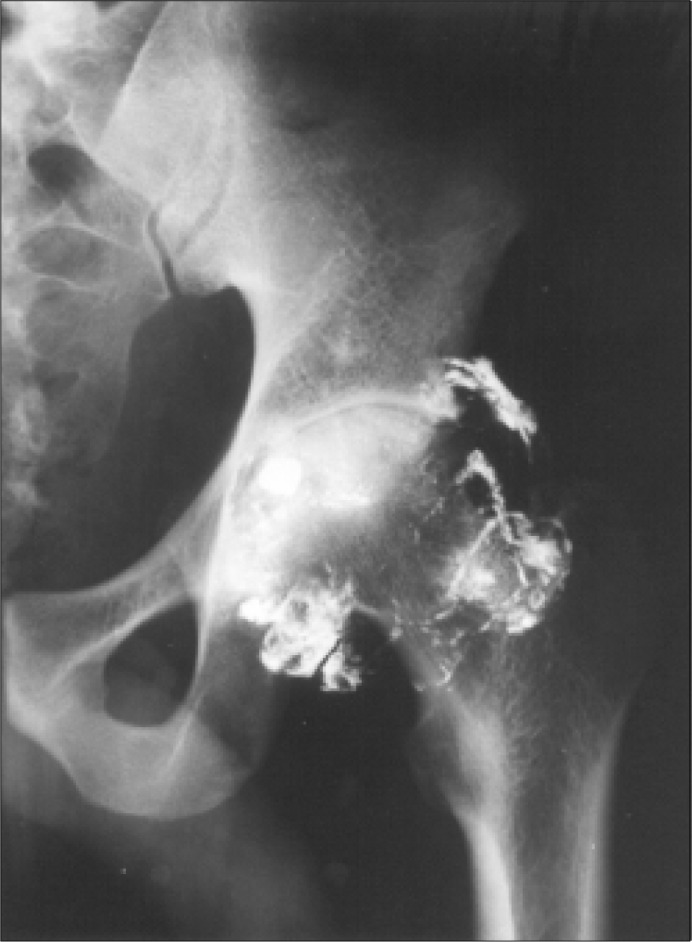
Radiograph of the left hip showing a fragment of the bullet and bursogram.

A course of chelating treatment using calcium versenate (EDTACaNa_2_) intravenously was started at a dosage of 1 g per day, and continued for 6 days. During the first hours of infusion of the specific treatment, the patient showed good improvement, being free from any abdominal pain by the second day of treatment. Chelation was assessed by measuring the total amount of lead excreted in urine during each day of treatment. The total amount of lead excreted in the 6 days was 20792 mg (mean 3465 mg each day), exceeding the cutoff of 2000 mg per day that is considered to be an efficacious treatment.

After the chelation therapy the patient had recurrence of his symptoms and a radical solution for the chronic mobilization of lead was considered. A hip arthroplasty procedure was performed, leading to a complete substitution of the left hip. Histopathologic examination of the hip and the part of the femur removed, showed intense metal impregnation with granulomatous foreign body reaction around the bone tissue and synovial capsule. Osteonecrotic areas with bone marrow fat saponification were also present, and disseminated calcification was noticed. Afterwards, during clinical follow-up, the patient no longer presented any symptoms related to lead intoxication.

## DISCUSSION

The present clinical case brings to light the importance of lead poisoning as a diagnosis for severe abdominal pain in emergency department units. Of course, occupational sources of exposure to lead are by far the most common ones, but retained bullets must be thought of as a possible source of lead if this is the case. The great number of people carrying lead bullets in some part of their bodies may increase the risk of "endogenous" intoxication by this heavy metal.

It has been observed that lead bullets in muscle or bone tissues, do not bring much trouble, as the tissue around the foreign body creates a fibrous capsule, avoiding dissolution of lead into the blood circulation^2^. Due to physical chemical properties, metallic lead tends to dissolve in acidic media, thereby promoting absorption, distribution, and toxic effects in its target organs and tissues, such as the central and peripheral nervous systems, the enzymatic system for heme synthesis, and kidneys. This is the case when the bullet is lodged in the joints, in direct contact with synovial liquid, or in the central nervous system or spinal cord canal, in contact with cerebrospinal fluid.^4^ In the case of big joints, like hips and shoulders, patients must not be lost to follow-up after emergency first care, and doctors must plan on early excision of the bullet, to avoid the risk of late dissolution and chronic clinical lead intoxication. The chronic synovial inflammatory process generally leads to great damage of the synovial capsule and joint cartilage surface, as seen in this case. Our case is very educational in this respect, as seven years elapsed between the shot accident and the surgical procedure that followed the diagnosis of intoxication. The patient's hip could probably have been spared if early intervention had been performed.

It is also worth mentioning the role of chelation therapy, with calcium versenate, or sodium EDTA, as a symptomatic and palliative treatment during the whole process of orthopedic and clinical evaluation before surgery. The great abdominal pain, and general symptom relief during chelation therapy, warrant the use of this procedure, not as a permanent treatment, as the source of endogenous lead is still there, but as a good way of improving the patient's clinical status in the lead-up to definite removal of the bullet.
